# The CentiMarker project: Standardizing quantitative Alzheimer's disease fluid biomarkers for biologic interpretation

**DOI:** 10.1002/alz.14587

**Published:** 2025-04-15

**Authors:** Guoqiao Wang, Yan Li, Chengjie Xiong, Yuchen Cao, Suzanne E. Schindler, Eric McDade, Kaj Blennow, Oskar Hansson, Jeffrey L. Dage, Clifford R. Jack, Charlotte E. Teunissen, Leslie M Shaw, Henrik Zetterberg, Laura Ibanez, Jigyasha Timsina, Cruchaga Carlos, the Alzheimer's Disease Neuroimaging Initiative, Randall J. Bateman

**Affiliations:** ^1^ Department of Neurology Washington University School of Medicine St. Louis Missouri USA; ^2^ Division of Biostatistics Washington University School of Medicine St. Louis Missouri USA; ^3^ Institute of Neuroscience and Physiology University of Gothenburg Mölndal Sweden; ^4^ Clinical Neurochemistry Laboratory Sahlgrenska University Hospital Mölndal Sweden; ^5^ Paris Brain Institute ICM Pitié‐Salpêtrière Hospital Sorbonne University Paris France; ^6^ Clinical Memory Research Unit Department of Clinical Sciences Malmö Faculty of Medicine Lund University Lund Sweden; ^7^ Memory Clinic Skåne University Hospital Malmö Sweden; ^8^ Department of Neurology Indiana University School of Medicine Indianapolis Indiana USA; ^9^ Department of Radiology Mayo Clinic Rochester Minnesota USA; ^10^ Neurochemistry Laboratory Department of Clinical Chemistry Amsterdam University Medical Centers Vrije Universiteit, Amsterdam Neuroscience Amsterdam Netherlands; ^11^ Department of Pathology & Laboratory Medicine University of Pennsylvania Perelman School of Medicine Philadelphia Pennsylvania USA; ^12^ Department of Neurodegenerative Disease UCL Institute of Neurology, Queen Square London UK; ^13^ UK Dementia Research Institute at UCL London UK; ^14^ Hong Kong Center for Neurodegenerative Diseases Hong Kong China; ^15^ Department of Psychiatry Washington University St. Louis Missouri USA; ^16^ Neuro Genomics and Informatics Washington University St. Louis Missouri USA

**Keywords:** Alzheimer's disease, biomarker standardization, CentiMarker, fluid biomarker

## Abstract

**INTRODUCTION:**

Biomarkers play a crucial role in understanding Alzheimer's disease (AD) pathogenesis and treatment effects. However, comparing biomarker measures without standardization and appreciating their magnitude relative to the disease can be challenging.

**METHODS:**

To address this issue, we propose the CentiMarker approach, similar to Centiloid, which provides a standardized scale between normal (0) and nearly maximum abnormal AD (100) ranges. We applied this scale to cerebrospinal fluid (CSF) biomarkers in dominantly inherited AD and sporadic AD cohorts.

**RESULTS:**

CentiMarkers facilitated the interpretation of disease abnormality, demonstrating comparable changes and distributions of AD biomarkers across disease stages. CentiMarkers make the treatment effect more comparable than their original scales across various biomarkers.

**DISCUSSION:**

The versatility of CentiMarkers makes it a valuable tool for standardized biomarker comparison in AD research, enabling informed cross‐study comparisons and contributing to accelerated therapeutic development. Adoption of the CentiMarker scale could enhance biomarker reporting and advance our understanding of AD.

**Highlights:**

- Comparing fluid biomarkers without appreciating their magnitude relative to the disease can be challenging.
- We propose a CentiMarker metric to standardize biomarker measures from normal (0) and nearly maximum abnormal AD (100) ranges.
- CentiMarkers make the treatment effect more comparable across various biomarkers than when using the original scales.

## 1 INTRODUCTION

The quantification and comparison of Alzheimer's disease (AD) biomarkers is crucial for understanding disease progression and the effectiveness of interventions. However, the current measures of fluid biomarker concentration, such as mass or moles per volume, are unique to each analyte, assay, and study.^1^, ^2^, ^3^, ^4^ This lack of standardization makes it challenging to compare results across different studies, laboratories, and biomolecules. Therefore, standardization efforts are crucial to allow for quantitative comparison of the AD biomarkers in the context of both biologic and treatment effects.

One kind of standardization is an absolute reference standard for the molar amount of analyte per volume unit. Current practice and requirements by health authorities advise or require standardization of assays based on the metrology approach with traceability back to the SI unit through certified reference materials (CRMs) and reference measurement procedures for diagnostic biomarkers for clinical use, as has been done for cerebrospinal fluid (CSF) amyloid beta 42 (Aβ42) by the International Federation of Clinical Chemistry (IFCC) Work Group.^5^ The final Aβ42 CRMs have also been used for re‐calibrating commercial immunoassays for CSF Aβ42, including those that are U.S. Food and Drug Administration (FDA) approved.^6^ However, this approach is very laborious and time‐consuming.

Other standardization methods, such as *z*‐score normalization, have been employed to facilitate comparisons of biomarkers within or across studies.^7^ *Z*‐score normalization typically involves utilizing a “control” group, such as a baseline or a reference group, like young healthy controls, to convert raw values or log‐transformed values into *z*‐scores. In this case, a 1 unit change in *z*‐score represents a 1 SD (standard deviation) change in the original scale. In our study, we aim to introduce an alternative, the concept of CentiMarkers, which is designed to transform fluid biomarker values onto a scale from 0 to 100. CentiMarkers aim to provide a common metric that allows for quantitative comparisons of AD biomarkers between normal and near maximum abnormal ranges. This would be helpful for comparing the same analyte and assay across different studies and cohorts. In addition, this would be helpful in comparing fluid biomarker values across different assays measuring the same analytes. For instance, the measurement of Aβ42/40 using different antibodies, immunoassays, or mass spectrometry methods can be standardized, thereby enabling meaningful comparisons both within and across studies.

Recent evidence from interventional trials suggests that some AD biomarkers that reach normal levels, such as achieving amyloid positron emission tomography (PET) negativity, have predictive value for clinical benefit.^2^, ^3^, ^8^ In addition, other biomarkers such as phosphorylated tau‐217 (p‐tau217) may be associated with cognitive impairment and response to treatment.^9^ However, comparing different disease cohorts, various forms of the disease, and diverse populations is often impractical due to the lack of a standardized biologic metric for fluid biomarker measurements. Having a common metric for these diverse analytes enables the interpretation of their biologic effects in relation to disease progression or response to interventions. Tracking disease progression or modification often involves monitoring multiple biomarkers, such as amyloid, tau, and neurodegeneration markers. The different AD biomarkers change sequentially over a 30‐year disease span (20 years before symptom onset through 10 years after symptom onset) and may not be monotonic.^10^, ^11^, ^12^, ^13^ Having a common metric for these diverse analytes enables the interpretation of their biologic effects in relation to disease progression or response to interventions.

The objectives of this study are the following: (1) to propose a mechanism for calculating CentiMarkers and showcase its applicability in different study cohorts; and (2) to illustrate the usefulness of CentiMarker values in facilitating the comparison of treatment effects across various fluid biomarkers for dominantly inherited AD (DIAD). This publication details the scope of use, methodology, contrasts approach for other purposes, and addresses potential limitations and explores alternative approaches. Future work will focus on how to generalize and apply the CentiMarker approach for other studies and purposes.

## 2 METHODS

### 2.1 Study oversight

The Dominantly Inherited Alzheimer Network Trial Unit 001 (DIAN‐TU‐001) study was conducted following the principles outlined in the Declaration of Helsinki and adhered to the guidelines set by the International Council for Harmonization and Good Clinical Practice. Ethical approval from the respective ethics committees at each participating site was obtained. Prior to participating in the study, all individuals provided written informed consent.

### 2.2 Study participants

DIAN‐TU‐001 is a randomized, placebo‐controlled, multi‐arm trial of gantenerumab or solanezumab in participants with DIAD across asymptomatic and symptomatic disease stages (NCT01760005). Mutation carriers (MCs) were assigned 3:1 to either drug or placebo and received treatment for 4–7 years. The information about these participants as well as the dosing schedule has been published in previous studies.^1^, ^4^ Briefly, the trial included 193 participants, consisting of 144 MCs and 49 non‐mutation carriers (NCs). The participants in the trial were either cognitively normal (Clinical Dementia Rating [CDR^©^ = 0]) or had early‐stage disease (CDR 0.5 or 1, indicating very mild or mild dementia) at the time of enrollment. The DIAN‐TU‐001 study presents a unique opportunity to utilize a subject set of young to middle‐aged healthy controls. These controls consist of family members of MCs without mutations (referred to as non‐mutation carriers or NCs). These NCs are young, healthy individuals without AD‐related disease pathologies.

**RESEARCH IN CONTEXT**

1. **Systematic review**: The authors conducted a thorough literature review, utilizing various sources including traditional platforms such as PubMed, as well as abstracts and presentations from scientific meetings. They identified and appropriately cited relevant publications related to the standardization of fluid biomarkers for Alzheimer's disease (AD).
2. **Interpretation**: Comparing biomarker measures for AD without standardization and understanding their significance in relation to the disease can be challenging. To address this issue, the authors propose the CentiMarker approach, akin to Centiloid, which establishes a standardized scale ranging from normal (0) to nearly maximum abnormal AD (100) ranges. The utilization of CentiMarkers facilitated the interpretation of disease abnormality, revealing comparable changes and distributions of AD biomarkers throughout different disease stages. Furthermore, CentiMarkers enable a more comparable assessment of treatment effects across various biomarkers compared to their original scales.
3. **Future directions**: Future research directions involve further testing of the CentiMarker approach in a broader range of studies, cohorts, and individual biomarkers to validate and refine its effectiveness. In addition, collaboration among different research groups will be vital in establishing bridging cohorts, similar to the approach used in Centiloid, to ensure harmonization and standardization across different studies and biomarker measurements.

DIAN Observational Study (DIAN‐OBS) enrolls participants with at least 50% risk of inheriting an DIAD mutation from families with a confirmed genetic mutation in *PSEN1*, *PSEN2*, or *APP*. At each study visit, participants underwent clinical assessments, cognitive testing, neuroimaging, and CSF biomarker collection. Specific information about the DIAN‐OBS can be found in previous publications.^11^, ^13^ DIAN‐OBS and DIAN‐TU‐001 follow similar protocols for clinical, cognitive, imaging, and biomarker measures. Only unique participant data from DIAN‐TU‐001 were included in this study, if participants were in both studies.

The Alzheimer's Disease Neuroimaging Initiative (ADNI) is a longitudinal study conducted in the United States focusing on sporadic AD and individuals at risk who undergo neuroimaging and biomarker evaluations. The study data utilized for our analysis ranged from 2008 to 2022. We specifically selected a subset of ADNI participants with derived estimated years from symptom onset (EYO), as reported in a previous study.^14^ Fluid biomarkers, which are common to all studies and have sufficient data, were converted into CentiMarkers to facilitate illustration and comparison.

### 2.3 Statistical methods

The parameters for calculating CentiMarker values in each of the three cohorts (DIAN‐TU‐001, DIAN‐OBS, and ADNI) were established using their respective data. Subsequently, in the re‐analysis of the DIAN‐TU‐001 trial, we interpreted the treatment effect of gantenerumab based on the CentiMarker unit, which provided a meaningful measure for assessing the efficacy across different fluid biomarkers.

#### 2.3.1 CentiMarker calculation

In order to convert the raw biomarker values to CentiMarker values, we utilized a methodology similar to that of the Centiloid conversion approach.^15^ For CentiMarker calculations, more abnormal biomarker raw values indicate worse disease stages as defined by the direction of disease versus normal groups. In summary, the process involves the following steps:

1. Identification of the CentiMarker‐0 (CM‐0) dataset and determination of the CM‐0 CentiMarker anchor value based on the CM‐0 cohort.
2. Identification of the CentiMarker‐100 (CM‐100) dataset and determination of the CM‐100 CentiMarker anchor value based on the CM‐100 cohort.
3. Calculation of CentiMarkers.

##### Identification of the CentiMarker‐0 dataset

To establish the CentiMarker‐0 anchor ($\mu_{\mathrm{CM}-0}$), all the data from the asymptomatic NCs enrolled in the DIAN‐TU‐001 trial are utilized. The first step involves calculating the interquartile range (IQR) of the data. Then, any outliers that fall outside the range of (>Q3+1.5×IQR) or (<Q1−1.5×IQR) are excluded from the dataset.^15^ Finally, the mean of the remaining data points was established as the CentiMarker‐0 anchor for subsequent CentiMarker calculations.

##### Identification of the CentiMarker‐100 dataset

To determine the CentiMarker‐100 anchor ($\gamma_{\mathrm{CM}-100}$), data from all MCs enrolled in the DIAN‐TU‐001 study who did not receive active treatments were used. This includes both the baseline data from participants who were assigned to the active treatment as well as the baseline and post‐baseline data from those who were assigned to placebo.

The same approach applied in the identification of the CentiMarker‐0 dataset is again employed to identify and remove outliers from this dataset. The CentiMarker‐100 anchor, denoted as $\gamma_{\mathrm{MC}-100}$, is established as the 95th percentile of the most abnormal value across the spectrum of disease stages in the CentiMarker‐100 dataset. With this approach, when the higher values indicate a more severe disease stage, the 95th percentile of the most abnormal value corresponds to the 95th percentile of the highest values; when the lower values indicate a more severe disease stage, the 95th percentile of the most abnormal value corresponds to the 5th percentile of the lowest values. The bootstrapping method was used to calculate the SD of the 95th percentile of the most abnormal value. When a biomarker demonstrates a monotonic disease progression trajectory, higher CentiMarkers consistently indicate a more severe disease stage, irrespective of the direction of its raw values. However, for biomarkers that do not exhibit a monotonic decline (e.g., decreasing after reaching a certain disease stage), higher values of the CentiMarker do not necessarily correspond to a more severe disease stage.

##### Calculation of CentiMarkers

The CentiMarker is calculated as:

(1) $$\mathrm{CM}\;=\frac{y-\mu_{\mathrm{CM}-0}}{\gamma_{\mathrm{CM}-100}-\mu_{\mathrm{CM}-0}}\;\times 100,$$

where y is the raw biomarker value, $\mu_{\mathrm{CM}-0}$ is the CentiMarker‐0 anchor, and $\gamma_{\mathrm{CM}-100}$ is the CentiMarker‐100 anchor.

#### 2.3.2 Benefit of using the same CentiMarker‐0 anchor cohort

Due to the two‐anchor approach involved in CentiMarker conversion, namely the 0 and 100 value, maintaining consistency in the CentiMarker‐0 subject set is crucial. This not only facilitates the comparability of CentiMarkers calculated using different CentiMarker‐100 anchors, but also makes them exchangeable. By keeping the CentiMarker‐0 cohort constant, we can utilize the same CentiMarker‐0 anchor ($\mu_{\mathrm{CM}-0}$) when calculating CentiMarker values regardless of how the $\gamma_{\mathrm{CM}-100}$ is determined. Assuming the CentiMarker is calculated using two different CentiMarker‐100 anchors as following:

$$\;CM_{1}=\frac{y-\mu_{\mathrm{CM}-0}}{\gamma_{\mathrm{CM}-100_{1}}-\mu_{\mathrm{CM}-0}}\;\times 100,$$

$$\;CM_{2}=\frac{y-\mu_{\mathrm{CM}-0}}{\gamma_{\mathrm{CM}-100_{2}}-\mu_{\mathrm{CM}-0}}\;\times 100,$$

where $\gamma_{\mathrm{CM}-100_{1}}$ and $\gamma_{\mathrm{CM}-100_{2}}$ are the two different CentiMarker‐100 anchors. Rearranging these two formulas, we have:

(2) $$CM_{2}=\frac{\gamma_{\mathrm{CM}-100_{1}}-\mu_{\mathrm{CM}-0}}{\gamma_{\mathrm{CM}-100_{2}}-\mu_{\mathrm{CM}-0}}\;\times CM_{1}.$$

Therefore, by using the same CentiMarker‐0 cohort, the method for determining the $\gamma_{\mathrm{CM}-100}$ is inherently exchangeable. In this study, we selected the 95th percentile of the most abnormal value as the CentiMarker‐100. CentiMarkers obtained through alternative methods, such as using the 90th percentile of the most abnormal value or the mean of all MCs, can be converted into each other using formula (2).

## 3 RESULTS

**FIGURE 1:**
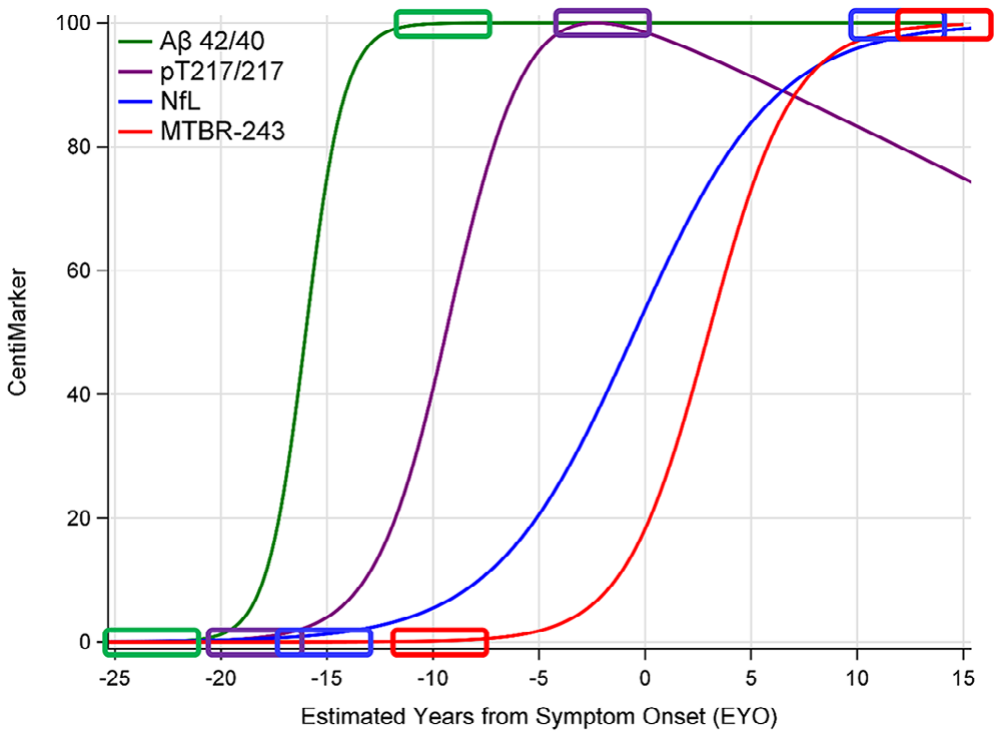
Illustration of CentiMarkers approach. The rectangles indicate the CentiMarker 0 and CentiMarker 100 for each biomarker. Aβ42/40, CSF Aβ42‐to‐40 ratio; pT217/217, CSF pT217‐to‐T217 ratio; NfL, CSF Neurofilament Light Chain; MTBR‐243, Microtubule Binding Region‐243.

Figure 1 illustrates the disease progression trajectories utilizing the CentiMarker concept. In this approach, each fluid biomarker is rescaled to a range of 0–100, enabling a straightforward, intuitive, and direct comparison across different biomarkers.

In addition, Table 1 provides the baseline demographics of all participants included in this study for each respective cohort.

**TABLE 1:** Baseline demographics for each cohort.

	DIAN‐TU‐001 MCs			DIAN‐OBS		ADNI		
	Active gantenerumab *N* = 52	Active solanezumab *N* = 50	Active placebo *N* = 40	Non‐mutation carriers *N* = 221	MCs *N* = 242	Controls *N* = 69	MCI group *N* = 335	AD group *N* = 160
Age (mean ± SD), years	46.0 ± 10.8	42.5 ± 9.5	44.2 ± 9.6	37.1 ± 11.0	36.3 ± 11.2	74.9 ± 5.4	73.3 ± 7.2	73.9 ± 8.0
Female, *n* (%)	21 (40)	29 (58)	22 (55)	106 (48)	128 (53)	37 (54)	130 (39)	67 (42)
Education (mean ± SD), years	14.8 ± 3.1	14.9 ± 2.9	15.5 ± 3.1	14.9 ± 2.8	14.4 ± 3.1	16.2 ± 2.6	15.9 ± 2.8	15.7 ± 2.7
CDR 0, *n* (%)	31 (60)	30 (60)	22 (55)	221 (100)	161 (67)	69 (100)	1 (0.3)	NA
CDR 0.5, *n* (%)	15 (29)	13 (26)	15 (38)	NA	44 (18)	NA	334 (100)	68 (43)
CDR 1, *n* (%)	6 (12)	7 (14)	3 (8)	NA	23 (10)	NA	NA	91 (57)
CDR ≥ 2, *n* (%)	NA	NA	NA	NA	14 (6)	NA	NA	1 (0.6)
CDR‐SB, *n* (%)	1.33 ± 2.08	1.37 ± 2.01	1.43 ± 1.87	0.01 ± 0.06	1.65 ± 3.49	0.07 ± 0.17	1.62 ± 0.89	4.52 ± 1.64
MMSE (mean ± SD)	27.10 ± 3.45	26.72 ± 4.11	26.68 ± 3.97	29.08 ± 1.24	26.21 ± 6.07	29.09 ± 1.04	27.58 ± 1.76	23.14 ± 2.09

Abbreviations: AD, Alzheimer's disease; ADNI, Alzheimer's Disease Neuroimaging Initiative; CDR, Clinical Dementia Rating; DIAN‐OBS, DIAN Observational Study; DIAN‐TU, Dominantly Inherited Alzheimer Network Trial Unit; MCs, mutation carriers; NA, not applicable.

### 3.1 Using the 95 percentile of the most abnormal value as the CentiMarker‐100 anchor

The two anchor values, CentiMarker‐0 and CentiMarker‐100, which were established using the DIAN‐TU‐001 data for each biomarker, are provided in Table S1. These values were then utilized in formula (1) to calculate the CentiMarkers for each biomarker accordingly.

**FIGURE 2 alz14587-fig-0002:**
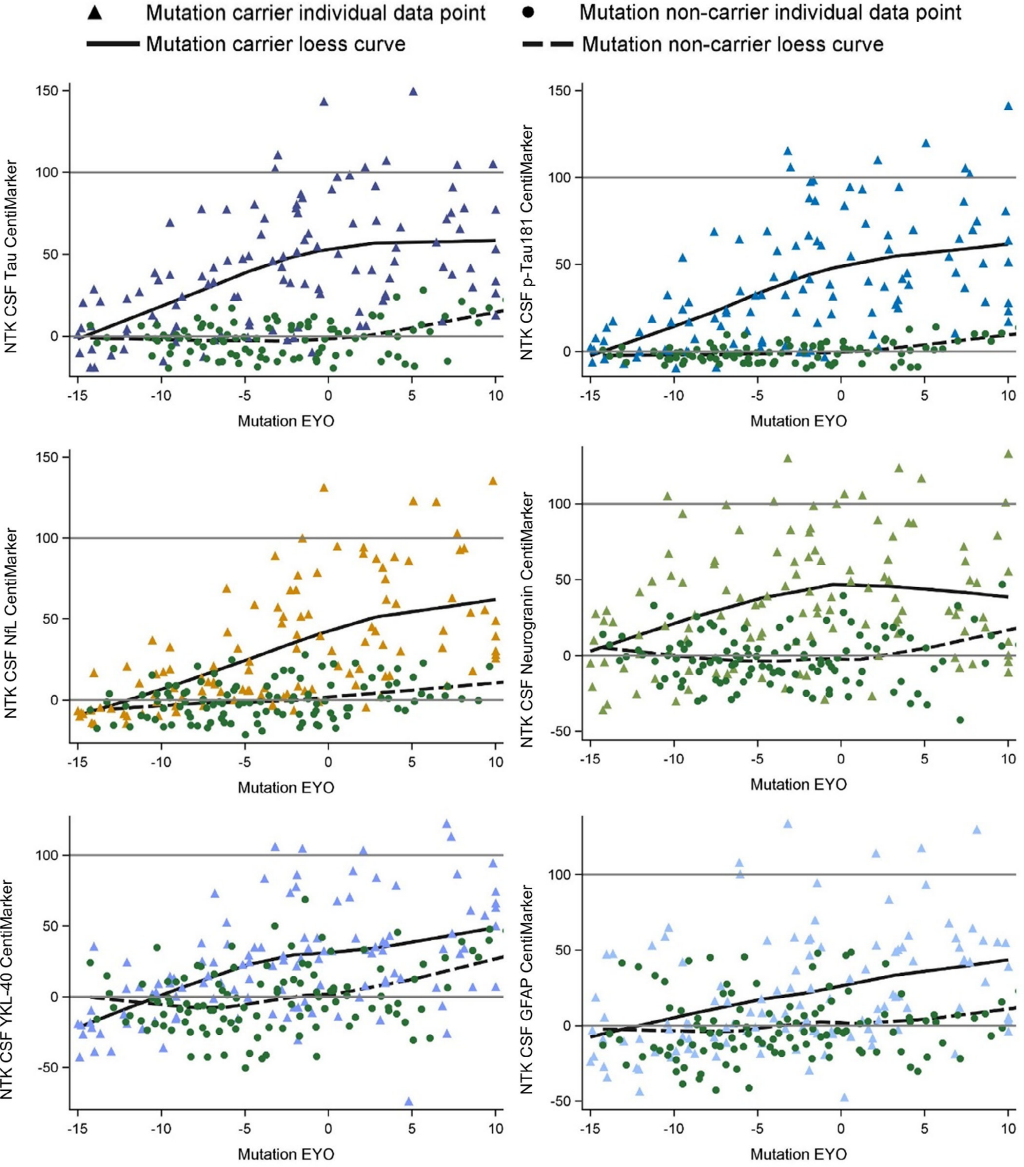
Comparison of different CentiMarkers in the same study by stage of disease between normal controls (green circles) and MCs (color triangles) as presented by the Loess curves and scatter data points. The MCs included only the baseline data from the treatment groups and all the data from the placebo group in the DIAN‐TU‐001 trial. The *x*‐axis represents the EYO based on mutation information, with zero indicating the expected onset of symptoms, covering a range of 25 years to represent disease progression. EYO, estimated years to symptom onset; MCs, mutation carriers.

Figure 2 illustrates the CentiMarkers for some of the fluid biomarkers published within the DIAN‐TU‐001 study.^16^ As anticipated, most CentiMarkers are observed to fall within the range of 0–100 and demonstrate an increasing trend as the disease progresses. Table S2 presents the means (SDs) of CentiMarkers for both the CentiMarker‐0 and CentiMarker‐100 datasets. As anticipated, the means for the CentiMarker‐0 dataset are all zero. In contrast, the means for the CentiMarker‐100 dataset change as the disease advances from asymptomatic (CDR 0) to symptomatic (CDR >0). Note that not all measures go up, and some measures are not monotonic, as they increase and then decrease as the disease advances (e.g., CSF tau).^11^, ^17^ By utilizing CentiMarkers in this way, the maximum and minimum abnormalities of the biomarker measures can be expressed on a common and simple scale to enable easy interpretation.^11^, ^18^

Figure S1 displayed standardized values from three different methods: CentiMarkers, Natural Log‐transformed CentiMarkers, and *Z*‐scores. Natural Log‐transformed CentiMarkers were calculated based on the natural log‐transformed values, whereas *z*‐scores were computed using the baseline mean and SD of the MCs. Despite the standardization method used, the general disease progression trajectories remain consistent, although the trajectory of the *z*‐score method tends to be lower compared to CentiMarkers.

### 3.2 Cross‐validation using DIAN‐OBS data

**FIGURE 3 alz14587-fig-0003:**
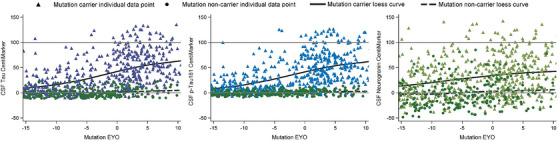
Comparison of different CentiMarkers as presented by the Loess curves and scatter data points within a cohort by stage of disease comparing normal controls (green circles) to MCs (color triangles) using the DIAN observational cohort. The *x*‐axis represents the EYO based on mutation information, with zero indicating the expected onset of symptoms, covering a range of 25 years to represent disease progression. EYO, estimated years to symptom onset; MCs, mutation carriers.

In order to assess the transferability of the CentiMarker method, we applied it to the DIAN‐OBS published data.^11^, ^12^ Figure 3 demonstrates the CentiMarker distribution of the four shared biomarkers between DIAN‐TU‐001 and DIAN‐OBS. NCs predominantly cluster around the zero line for all biomarkers. MCs exhibit a similar pattern and dispersion to those observed in DIAN‐TU‐001, with the majority of data points within the range of 0–100.

**FIGURE 4 alz14587-fig-0004:**
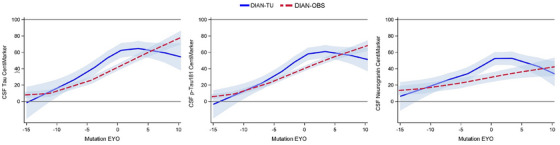
Comparison of CentiMarkers across clinical cohorts utilizing disease progression trajectories between DIAN‐TU‐001 (non‐treated, blue solid line) and DIAN‐OBS (observational cohort in red dashed line) as presented by the Loess curves with 95% CI. These curve fits show similar biomarker changes by EYO across studies demonstrating comparability of CentiMarkers across studies. EYO, estimated years to symptom onset.

Figure 4 presents a comparison between DIAN‐TU‐001 (non‐treated placebo and baseline) and DIAN‐OBS in CentiMarkers across the entire disease progression for MCs. Despite these two different studies, their disease progression trajectories closely resemble each other, as shown in Figure 4.

### 3.3 Treatment effect in CentiMarkers

**FIGURE 5 alz14587-fig-0005:**
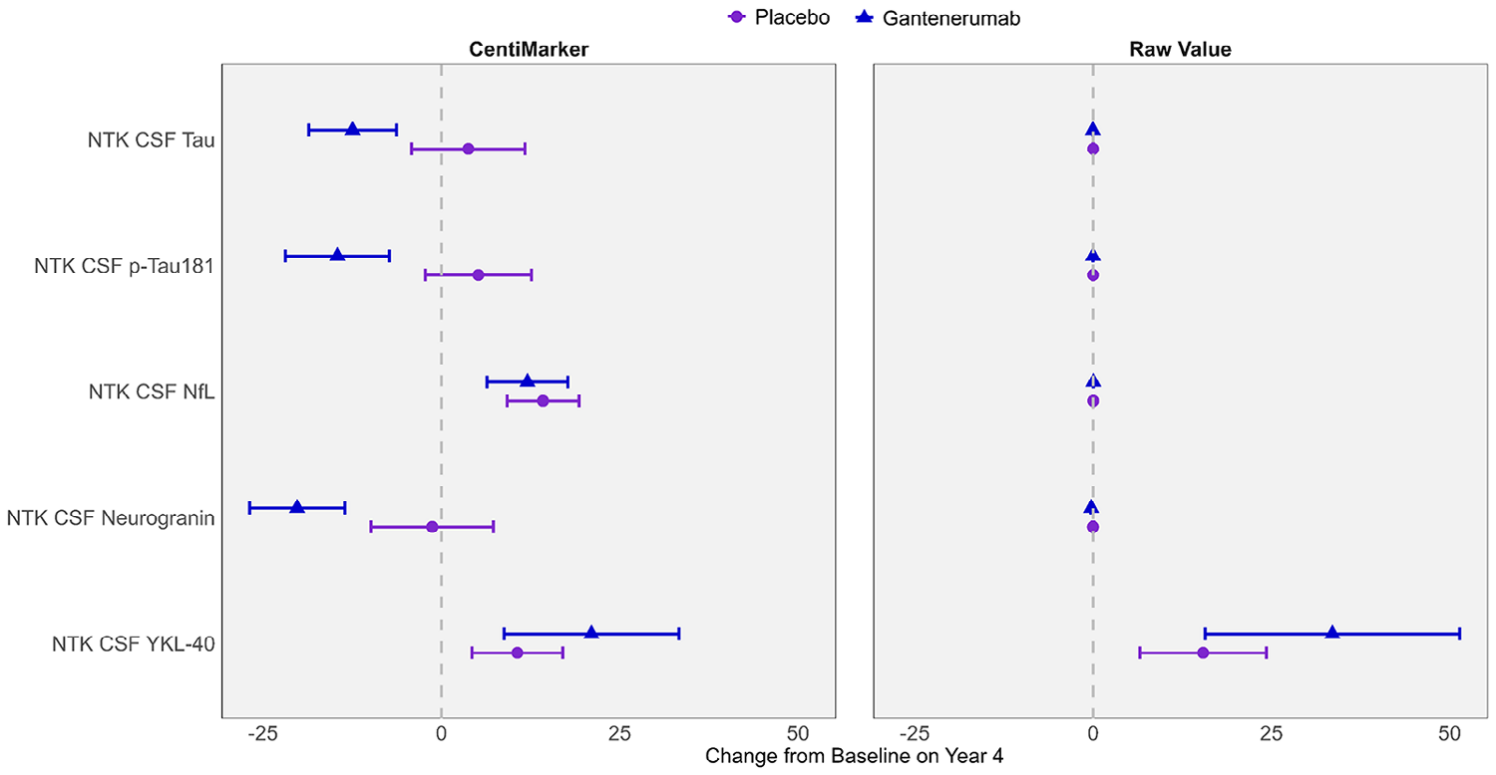
Utilizing CentiMarkers facilitates the interpretation and comparison across biomarkers by converting them to a similar scale, whereas using raw values is more difficult to compare as units are different and not scaled to disease ranges. Estimated mean change from baseline in CentiMarkers with 95% confidence intervals for the gantenerumab and placebo groups using MMRM analyses in the DIAN‐TU‐001 trial. These results demonstrate the magnitude of disease normalization compared to normal (CM 0) versus fully abnormal (CM 100) states. Raw values are in the unit of ng/mL.

Figure 5 illustrates the change from baseline to Year 4 for both the gantenerumab and the placebo groups as measured in both CentiMarker units and in raw values (as published previously^16^). CentiMarkers facilitate the interpretation and comparison of the treatment effect compared to the raw values. The changes from baseline to Year 4 in both the gantenerumab and the placebo groups are comparable in CentiMarkers across all the biomarkers, which contrasts with those using raw values.^16^ Figure S2 illustrates the comparison, including glial fibrillary acidic protein (GFAP). It is worth noting that GFAP exhibits larger variability in the gantenerumab group, making it less comparable to other biomarkers. However, the change observed in the placebo group remains consistent across all biomarkers, including GFAP.

Figure S3 illustrates the change from baseline over a 4‐year treatment period. The CentiMarkers clearly depict the distance of participants from the normal level at baseline and the extent to which the treatment has brought them closer to the normal level after 4 years. For instance, when considering CSF tau, the administration of gantenerumab resulted in a decrease of 12 units in CentiMarkers, reducing it from 46 to 34. However, it is important to note that even at the end of the treatment, participants still remained 34 units above the normal CentiMarker value. Table S3 presents a comparison of statistical significance between CentiMarkers and the original raw values. Because CentiMarkers are a linear transformation of the original values, the statistical significance is identical.[Fig alz14587-fig-0002], [Fig alz14587-fig-0003], [Fig alz14587-fig-0004], [Fig alz14587-fig-0005]

**FIGURE 6:**
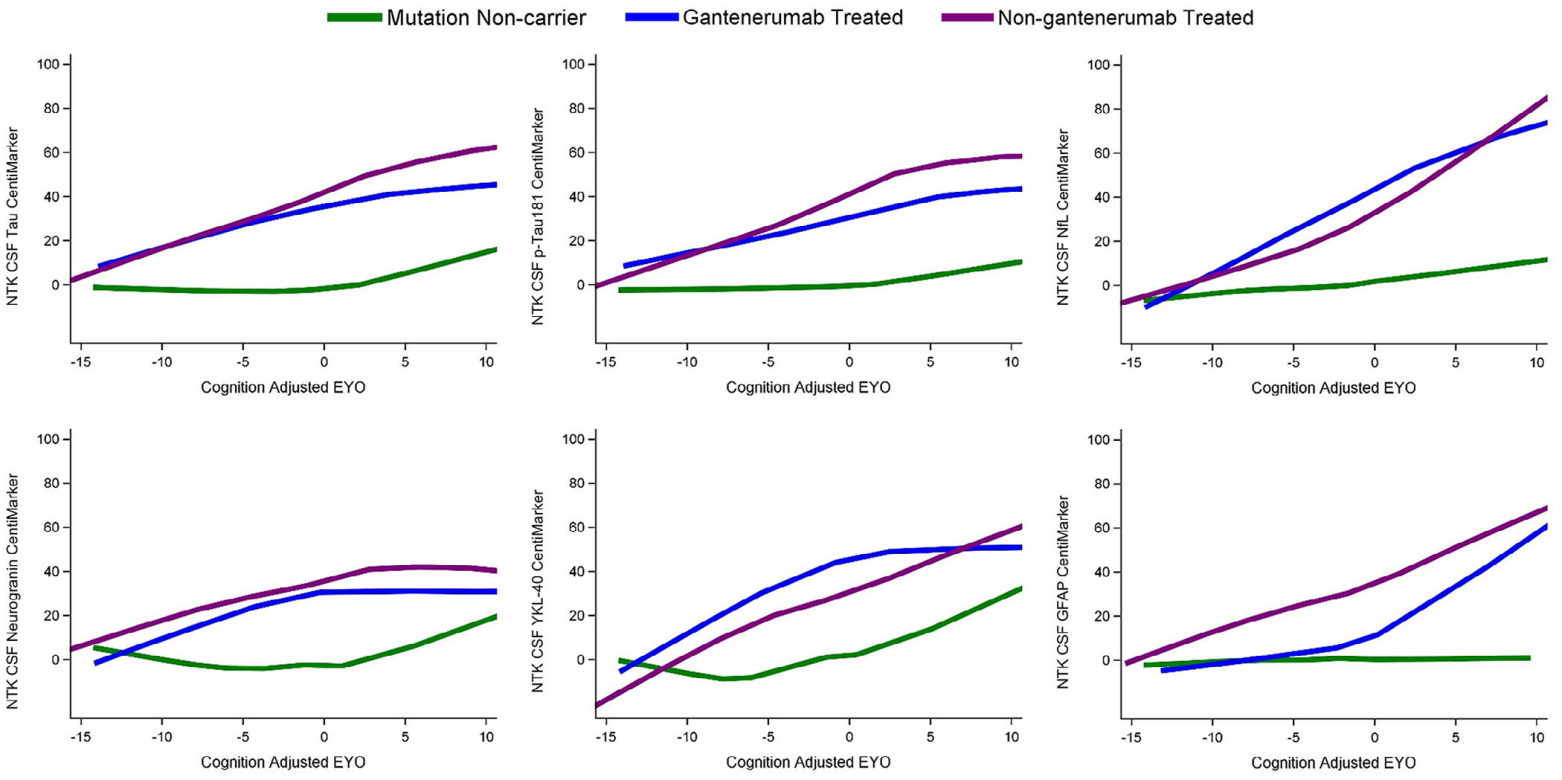
Illustration of treatment effects over EYO relative to mutation non‐carriers (green line). Participants treated with gantenerumab (blue line) showed slower progression than those non‐gantenerumab treated (purple line) across multiple biomarkers as presented by the Loess curves with 95% CI. The relative differences of treatment effect indicate that although there were improvements in multiple biomarkers, the measures did not reach normal CentiMarker levels for some measures. EYO, estimated years to symptom onset.

Figure 6 illustrates the comparison of disease progression for each biomarker across a time span of 15 years preceding symptom onset to 10 years following symptom onset. This comparison included three groups: non‐mutation carriers (or NCs; i.e., cognitively normal individuals), MCs treated with gantenerumab, and MCs not receiving gantenerumab treatment. CentiMarkers allow us to quickly identify biomarkers that exhibit a similar pattern of progression and evolve at a similar rate.

### 3.4 CentiMarkers in ADNI

**FIGURE 7:**
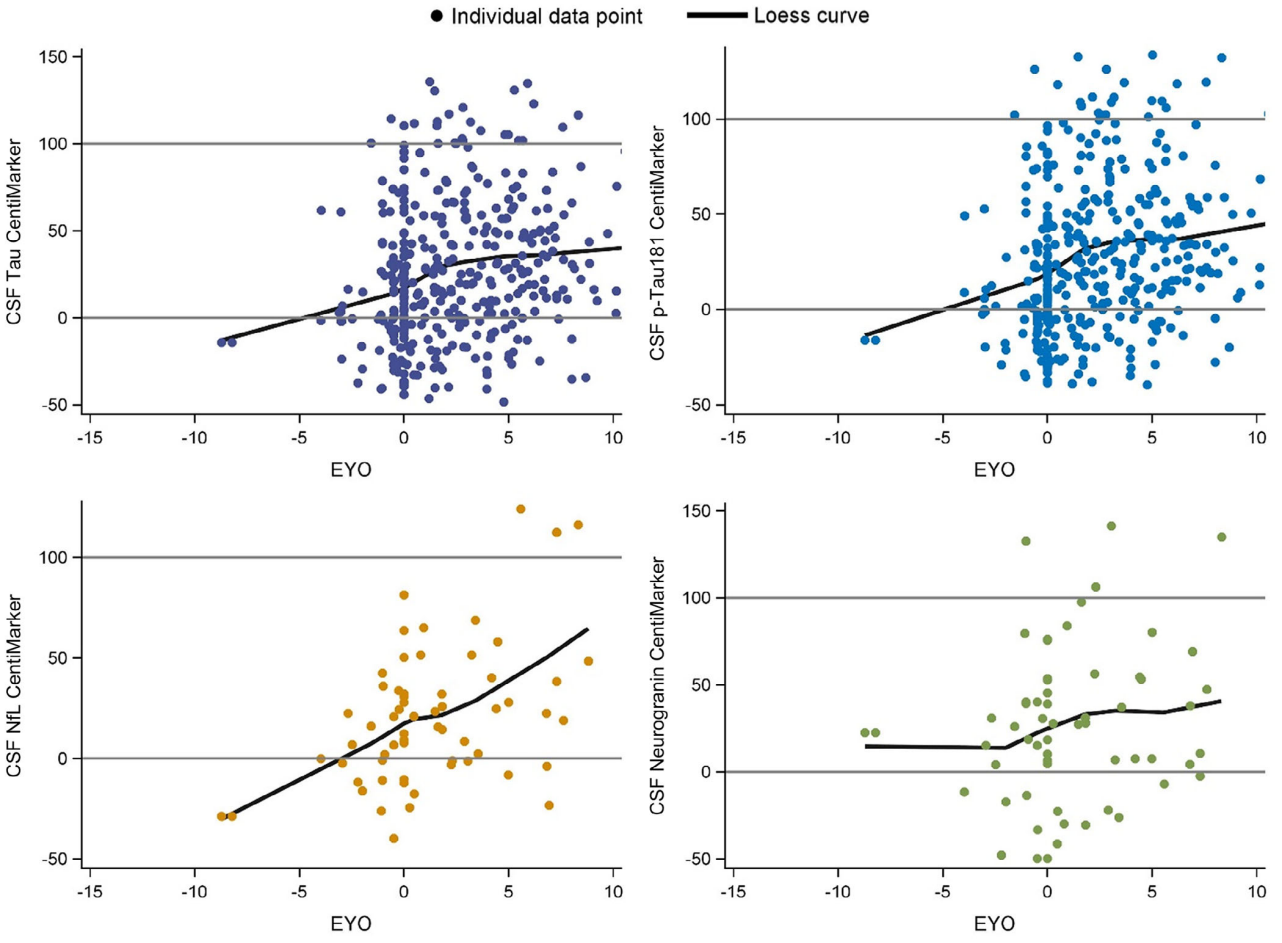
Illustration of CentiMarkers in ADNI as presented by the Loess curves and scatter data points. EYO is calculated by using the individual's own age at symptom onset as EYO of 0. ADNI, Alzheimer's Disease Neuroimaging Initiative; CSF, cerebrospinal fluid. EYO, estimated years to symptom onset.

In the ADNI study, the calculation of CentiMarker 0 (normal cohort) incorporated datasets from the clinical normal cohort. This cohort was defined as individuals with a CDR score of 0, indicating no significant cognitive impairment. In addition, participants in this cohort had a diagnosis status of cognitive normal and fell within the age range of 50–89 years of age. The CentiMarker 100 cohort in the study consisted of individuals with cognitive impairment, specifically those with a CDR score greater than 0. This cohort included individuals diagnosed with mild/moderate cognitive impairment or AD and had an age range of 50–89 years. The CentiMarker‐0 (mean (SD)) and CentiMarker‐100 (95th percentile (SD)) for CSF total tau are 240.0  (80.8) (pg/mL) and 527.5 (8.4) (pg/mL); 21.6  (7.7) (pg/mL) and 54.3 (1.0) (pg/mL) for CSF p‐tau181; 1051.0 (348.3) (ng/L) and 2411.0 (153.4) (ng/L) for CSF NfL; and 315.4 (199.8) (pg/mL) and 948.3 (53.7) (pg/mL) for CSF neurogranin. Figure 7 illustrates the CentiMarkers of the four shared fluid biomarkers collected in both the DIAN‐TU‐001 and ADNI studies. Similar to the findings in the DIAN studies, the CentiMarkers in the ADNI study mostly fall below 100 and tend to cluster within the range of 0–100.

## 4 DISCUSSION

AD researchers have developed biomarkers of AD pathogenesis, pathophysiology, progression, and treatment effects that have become among the most advanced in both breadth and accuracy of common diseases. However, because each biomarker measure is a representation of the biological target, the assay used to measure it, and the variance of the assay, these biomarker measures are difficult to compare between biomarkers, across studies, and even within a biomarker type (e.g., Aβ or p‐tau concentrations). AD is a complex neurodegenerative disease characterized by the accumulation of Aβ plaques and tau tangles in the brain with associated inflammation and neuronal injury. However, the relationship between the magnitude of change in biomarkers and the clinical symptom of AD is not linear, making it challenging to interpret the disease progression and the effects of potential interventions.

### 4.1 Rationale for establishing CentiMarkers

We have developed a conceptual framework to facilitate the interpretation of biomarker changes in AD studies. This approach, called CentiMarkers, aims to mathematically transform biomarker measurements onto a standardized scale, with 0 representing normal and 100 representing nearly (95%) maximally abnormal levels, conceptionally similar but not identical to Centiloid scales used in PET imaging.^15^ This transformation addresses several challenges in AD biomarker research.

First, it provides a more intuitive measure of the magnitude of biomarker change. This is essential for both observational studies and clinical trials, facilitating comparisons between drugs and their effects at different stages of the disease and the comparison of stage of disease across cohorts. Second, this approach partly addresses the lack of comparability across biomarker assays. Although no standardization can provide cross‐assay equivalence, it enables a reasonable approximation for understanding overall trends in biomarker dynamics. It is important to note that this concept was developed through extensive discussion and collaboration with experts across research groups in biomarkers, imaging, clinical studies, and interventional trials. This article introduces the CentiMarker method, offering a conceptual framework and practical application for its use in observational studies and interventional trials. The method is demonstrated using large AD cohorts from the ADNI and DIAN studies, providing real‐world examples. We anticipate that this approach will enhance the communication of AD biomarker results, making them more readily understood by the broader research community.

### 4.2 Defining normal and abnormal ranges for normalization

The CentiMarker 0 is the mean value in a normal cohort, defined by each study. The CentiMarker approach uses the 95th percentile most abnormal value (i.e., the value defining the most abnormal 5% of the affected population) to define the CentiMarker 100; this is different from amyloid PET Centiloid, which uses the mean of individuals with early symptomatic AD to define Centiloid 100. Unlike amyloid PET, which has a monotonic change until very late in the course of AD, fluid biomarkers may reach their maximum earlier in the course of AD, and importantly reach maximums at different stages of disease (see Figure 1). Therefore, CentiMarker 100 is defined by the 95th percentile because of the more complex trajectories of these biomarkers. By establishing CentiMarker 100 as the upper abnormal value for biomarkers across the entire range of disease stages and time, a maximum abnormal upper range is set, irrespective of when and how the biomarker changes or whether it exhibits non‐monotonic behavior. In cases where only a small cohort is available for deriving the CentiMarker 100 value or concerns exist regarding nonrepresentative outliers, an alternative approach can be implemented. Rather than relying on the maximum upper range, the CentiMarker 100 value can be determined using either the 90th percentile abnormal value within the available cohort or by referencing the CentiMarker 100 value reported in other studies utilizing the same assay.

To derive the CentiMarker scale,^15^ we recommend having a minimum of 30 data points for the CentiMarker 0 group in order to obtain a relatively accurate estimation of the mean. Through extensive bootstrapping estimation, our analysis suggests that having 30–50 data points for the CentiMarker 100 groups can yield a relatively stable value for the 95th most abnormal value. Notably, 50%–70% of the bootstrapped 95th most abnormal values fall within 10% of the overall group's 95% most abnormal value, varying depending on the biomarkers’ variability.

Another issue is that as the disease advances, some biomarkers (e.g., amyloid PET) may plateau, but other biomarkers (e.g., brain atrophy) are likely to continue to progress, even after participants are too impaired to participate in studies, thus precluding the defining of the full disease course for CentiMarker 100. Future work would need to address this for studies that extend beyond defining CentiMarker‐0 and CentiMarker‐100 groups.

### 4.3 Use of CentiMarkers

CentiMarkers offer a solution for ensuring comparability across different biomarker measures and units, as well as accounting for disease effects. By transforming biomarker measurements onto a common scale ranging from normal to maximally abnormal, CentiMarkers provide a standardized framework for accurate comparison and assessment. This allows for a more intuitive understanding of the magnitude of biomarker changes and their relationship with the clinical symptoms of AD. For instance, a CentiMarker value of 50 would indicate that a biomarker is halfway between the normal and maximum abnormal ranges, providing a clear and straightforward interpretation of the biomarker status. Moreover, CentiMarkers can be used to compare the effects of different interventions on AD biomarkers. This is particularly important in the context of clinical trials, where multiple biomarkers are often assessed simultaneously. For instance, consider a scenario where a CentiMarker originally measured 50 and after treatment, it decreased to 0. In this case, the biological measure abnormality has been effectively corrected. On the other hand, if the CentiMarker decreased from 100 to 50, despite a similar magnitude of change, the measure has not been biologically corrected. By providing a common metric for all biomarkers, CentiMarkers allow us to differentiate between treatments that fully normalize the biological measure and those that only produce partial improvement, and also facilitate direct comparisons of the effects of different biological effects.

### 4.4 Future research and application

The application of CentiMarkers is not limited to AD research. The concept can be generalized to other neurodegenerative diseases, such as Parkinson's disease and Huntington's disease. This broad applicability makes CentiMarkers a potentially powerful tool for effective communication of biomedical research results.

### 4.5 Challenges and limitations

However, the implementation of CentiMarkers also has its challenges. It requires a well‐characterized disease cohort and a normal control to define CentiMarkers. Each assay must be tested on these cohorts, which can be resource intensive. Furthermore, different ways to choose maximum and minimum abnormality may lead to different scales, thereby limiting comparisons. Other harmonization techniques, like the use of *z*‐scores, have been employed to facilitate direct comparisons across fluid biomarkers. However, it is important to note that the *z*‐score approach does not incorporate disease normality into its scale. In addition, a 1‐unit change in *z*‐score corresponds to a 1 SD change in the original raw values. Therefore, the comparability of *z*‐scores relies on measurement variability, which may be influenced by factors such as assay precision and biological variation. Other efforts utilize certified reference standards to make individual assays or analytes comparable across studies, but this only works within an analyte, not across analytes, and again does not provide relative disease information. The CentiMarker is a linear transformation of the raw values, so it does not alter statistical significance. Consequently, the statistical interpretation of a significant test based on CentiMarker values remains the same as it would be with raw values. The primary advantage of the CentiMarker is its intuitive representation of treatment effects across disease stages and biomarkers. For example, a 10‐point reduction in CentiMarker signifies a treatment effect that shifts patients 10 points closer to a biomarker's normal, regardless of the specific biomarker being assessed. And this reduction may hold greater clinical significance at a baseline CentiMarker value of 30 compared to 80. In summary, CentiMarkers offer several advantages over typical log‐transformed or *z*‐standardized values: (1) they preserve the statistical significance observed with raw values, whereas log‐transformed or *z*‐standardized values can sometimes yield different, often more significant, test results; (2) thresholds or cutoffs established from raw values can be directly converted into CentiMarker units; and (3) unlike log transformation or *z*‐standardization, CentiMarkers maintain the original disease progression trajectories estimated from raw data.

Future research directions include testing the approach in more studies, cohorts, and individual biomarkers to validate and refine the method.^7^ In addition, collaboration among various research groups will be crucial in establishing bridging cohorts similar to the Centiloid approach.^19^ This collaborative effort aims to facilitate the conversion of CentiMarker measurements across various cohorts and assays, thereby enhancing comparability across different fluid biomarkers. An important future goal and challenge will be to ensure researcher understanding, uptake, and utilization of CentiMarkers in their studies. The coordination and harmonization of specific approaches are necessary to enable the comparability of findings. With these combined efforts, CentiMarkers have the potential to revolutionize our understanding of AD and other diseases, thereby expediting the development of effective treatments.

## CONFLICT OF INTEREST STATEMENT

G.W.is the biostatistics core co‐leader for the Dominantly Inherited Alzheimer Network‐Trial Unit (DIAN‐TU).^19^ He discloses serving on the Data Safety Monitoring Board (DSMB) for Eli Lilly and Company, Amydis Corporate, and Abata Therapeutics, as well as working as a statistical consultant for Alector, Inc. and Pharmapace, Inc. He also serves as DSMB member for another five studies funded by the National Institutes of Health (NIH). R.J.B.is the Director of the DIAN‐TU and Principal Investigator of the DIAN‐TU‐001. He co‐founded C2N Diagnostics. Washington University and R.J.B. have equity ownership interest in C2N Diagnostics and receive royalty income based on technology (stable isotope labeling kinetics, blood plasma assay, and methods of diagnosing Alzheimer's disease [AD] with phosphorylation changes) that is licensed by Washington University to C2N Diagnostics. R.J.B. receives income from C2N Diagnostics for serving on the scientific advisory board. R.J.B. has received research funding from Avid Radiopharmaceuticals, Janssen, Roche/Genentech, Eli Lilly, Eisai, Biogen, AbbVie, Bristol Myers Squibb, and Novartis. He receives research support from the National Institute on Aging (NIA) of the NIH, DIAN‐TU Trial Pharmaceutical Partners (Eli Lilly and Company, F. Hoffman‐La Roche, Ltd., and Avid Radiopharmaceuticals), Alzheimer's Association, GHR Foundation, Anonymous Organization, DIAN‐TU Pharma Consortium (Active: Biogen, Eisai, Eli Lilly and Company, Janssen, F. Hoffmann‐La Roche, Ltd./Genentech. Previous: AbbVie, Amgen, AstraZeneca, Forum, Mithridion, Novartis, Pfizer, Sanofi, and United Neuroscience). He has been an invited speaker for Novartis and serves on the Advisory Board for F. Hoffman La Roche, Ltd. E.M.is the Associate Director of the DIAN‐TU. He reports serving on a Data Safety Committee for Eli Lilly and Company and Alector; scientific consultant for Eisai and Eli Lilly and Company; institutional grant support from Eli Lilly and Company, F. Hoffmann‐La Roche, Ltd., and Janssen. J.L.D. is an inventor on patents or patent applications of Eli Lilly and Company relating to the assays, methods, reagents and/or compositions of matter for p‐tau assays and Aβ‐targeting therapeutics. J.L.D. has served as a consultant on advisory boards for Eisai, Abbvie, Genotix Biotechnologies Inc, Gates Ventures, Karuna Therapeutics, AlzPath Inc., Cognito Therapeutics, Inc., and Prevail Therapeutics, and received research support from ADx Neurosciences, Fujirebio, AlzPath Inc., Roche Diagnostics, and Eli Lilly and Company in the past 2 years. J.L.D. has received speaker fees from Eli Lilly and Company. J.L.D. is a founder and advisor for Monument Biosciences. J.L.D. has stock or stock options in Eli Lilly and Company, Genotix Biotechnologies, AlzPath Inc., and Monument Biosciences. S.E.S. has served as a consultant or an advisory board or received speaker's fees from Eisai, Eli Lilly, and Novo Nordisk. K.B. has served as a consultant and at advisory boards for Abbvie, AC Immune, ALZPath, AriBio, BioArctic, Biogen, Eisai, Lilly, Moleac Pte. Ltd, Neurimmune, Novartis, Ono Pharma, Prothena, Roche Diagnostics, and Siemens Healthineers; has served on data monitoring committees for Julius Clinical and Novartis; has given lectures, produced educational materials, and participated in educational programs for AC Immune, Biogen, Celdara Medical, Eisai, and Roche Diagnostics; and is a co‐founder of Brain Biomarker Solutions in Gothenburg AB (BBS), which is a part of the GU Ventures Incubator Program, outside the work presented in this article. H.Z. has served at scientific advisory boards and/or as a consultant for Abbvie, Acumen, Alector, Alzinova, ALZPath, Amylyx, Annexon, Apellis, Artery Therapeutics, AZTherapies, Cognito Therapeutics, CogRx, Denali, Eisai, LabCorp, Merry Life, Nervgen, Novo Nordisk, Optoceutics, Passage Bio, Pinteon Therapeutics, Prothena, Red Abbey Labs, reMYND, Roche, Samumed, Siemens Healthineers, Triplet Therapeutics, and Wave; has given lectures in symposia sponsored by Alzecure, Biogen, Cellectricon, Fujirebio, Lilly, Novo Nordisk, and Roche; and is a co‐founder of Brain Biomarker Solutions in Gothenburg AB (BBS), which is a part of the GU Ventures Incubator Program (outside the submitted work). C.C. has received research support from GSK and Eisai. The funders of the study had no role in the collection, analysis, or interpretation of data; in the writing of the report; or in the decision to submit the paper for publication. C.C. is a member of the advisory board of Circular Genomics and owns stocks in this company. All the other authors reported no conflicts of interest. Author disclosures are available in the Supporting Information.

## CONSENT STATEMENT

The DIAN‐TU study was conducted in accordance with the Declaration of Helsinki and the International Council for Harmonization and Good Clinical Practice guidelines and had ethics committee approval at each participating site. All participants provided written informed consent.

## Supporting information



Supporting Information

Supporting Information
