# Supplementary material for: The CentiMarker project: Standardizing quantitative Alzheimer's disease fluid biomarkers for biologic interpretation
Source: Alzheimers Dement. 2025 Apr 15;21(4):e14587. doi: 10.1002/alz.14587 (PMC12000244; doi:10.1002/alz.14587)
Supplement: Supplementary file 1 — Supporting Information [file ALZ-21-e14587-s001.docx]

**Supplemental Materials**

**Supplemental Table 1:** CentiMarker-0 and CentiMarker-100 values for each biomarker within the DIAN-TU-001 population

| Biomarker | CentiMarker 0 values (Mean ± SD (n)) of NMCs | CentiMarker 100 values (95 Percentile of the Most Abnormal Score ± SD (n)) of MCs |
| --- | --- | --- |
| NTK CSF Tau (pg/mL) | 135.20 ± 30.87 (109) | 399.90 ± 7.56 (198) |
| NTK CSF p-Tau181 (pg/mL) | 11.95 ± 2.45 (93) | 52.93 ± 1.51 (195) |
| NTK CSF Neurofilament Light Chain Protein (pg/mL) | 67.69 ± 20.33 (113) | 242.60 ± 9.71 (204) |
| NTK CSF Neurogranin (pg/mL) | 842.82 ± 223.63 (119) | 2066.00 ± 67.03 (206) |
| NTK CSF YKL-40 Protein (ng/mL) | 105.35 ± 33.71 (117) | 247.20 ± 8.73 (206) |
| NTK CSF Glial Fibrillary Acidic Protein (ng/mL) | 5.14 ± 1.88 (116) | 13.75 ± 0.78 (210) |
| DIAN OBS CSF Tau | 238.73 ± 68.86 (324) | 1048.00 ± 37.36 (650) |
| DIAN OBS CSF p-tau181 | 25.75 ± 8.00 (371) | 190.80 ± 7.43 (686) |
| DIAN OBS CSF Neurogranin (pg/mL) | 1527.93 ± 808.03 (356) | 4383.95 ± 111.21 (635) |

**Supplemental Table 2:** Mean ± SD (N) of CentiMarkers for the CentiMarker-0 dataset and CentiMarker-100 dataset by CDR global for the DIAN-TU-001 cohort

| Biomarker | CM-0 (NMCs), mean±SD (N) | CM-100 (MCs), mean±SD (N) | | | |
| --- | --- | --- | --- | --- | --- |
|  | CDR = 0 | CDR = 0 | CDR = 0.5 | CDR = 1 | CDR >= 2 |
| NTK CSF Tau (pg/mL) | 0.00 ± 11.66 (109) | 25.31 ± 28.50 (119) | 55.81 ± 34.89 (56) | 68.39 ± 32.60 (18) | 42.37 ± 49.43 (5) |
| NTK CSF p-Tau181 (pg/mL) | 0.00 ± 5.98 (93) | 22.95 ± 26.73 (116) | 51.89 ± 31.01 (55) | 68.82 ± 33.99 (19) | 28.50 ± 35.27 (5) |
| NTK CSF neurofilament light chain (pg/mL) | 0.00 ± 11.62 (113) | 12.35 ± 22.32 (122) | 53.16 ± 29.78 (62) | 86.01 ± 38.24 (17) | 55.61 ± 34.39 (3) |
| NTK CSF Neurogranin (pg/mL) | 0.00 ± 18.28 (119) | 24.77 ± 35.08 (123) | 44.70 ± 36.54 (60) | 52.11 ± 32.45 (18) | 11.74 ± 50.52 (5) |
| NTK CSF YKL-40 Protein (ng/mL) | 0.00 ± 23.77 (117) | 13.04 ± 31.27 (122) | 36.14 ± 37.99 (61) | 60.50 ± 38.72 (18) | 50.86 ± 45.53 (5) |
| NTK CSF Glial Fibrillary Acidic Protein (ng/mL) | 0.00 ± 21.81 (116) | 9.50 ± 32.89 (123) | 34.88 ± 39.61 (62) | 37.15 ± 28.95 (20) | 38.23 ± 58.91 (5) |

**Supplemental Table 3:** Illustration of identical statistical significance between raw values and CentiMarker values.

| Variable name | Method | Estimate | SE | DF | t Value | Pr > \|t\| |
| --- | --- | --- | --- | --- | --- | --- |
| NTK CSF Tau | CentiMarker | -12.48 | 3.01 | 116 | -4.14 | <.0001 |
| NTK CSF Tau | Raw Value | -33.04 | 7.97 | 116 | -4.14 | <.0001 |
| NTK CSF pTau | CentiMarker | -14.20 | 3.56 | 112 | -3.99 | 0.0001 |
| NTK CSF pTau | Raw Value | -5.82 | 1.46 | 112 | -3.99 | 0.0001 |
| NTK CSF NfL | CentiMarker | 21.52 | 4.38 | 122 | 4.91 | <.0001 |
| NTK CSF NfL | Raw Value | 37.63 | 7.67 | 122 | 4.91 | <.0001 |
| NTK CSF Neurogranin | CentiMarker | -20.34 | 3.27 | 120 | -6.22 | <.0001 |
| NTK CSF Neurogranin | Raw Value | -248.75 | 39.99 | 120 | -6.22 | <.0001 |
| NTK CSF YKL-40 | CentiMarker | 21.60 | 6.03 | 122 | 3.58 | 0.0005 |
| NTK CSF YKL-40 | Raw Value | 30.64 | 8.55 | 122 | 3.58 | 0.0005 |
| NTK CSF GFAP | CentiMarker | 73.59 | 68.89 | 123 | 1.07 | 0.2875 |
| NTK CSF GFAP | Raw Value | 6.34 | 5.93 | 123 | 1.07 | 0.2875 |
| Estimated treatment effects at the Year 4 visit are presented for illustrative purposes. Similar results are observed at the other visits as well. | | | | | | |

**
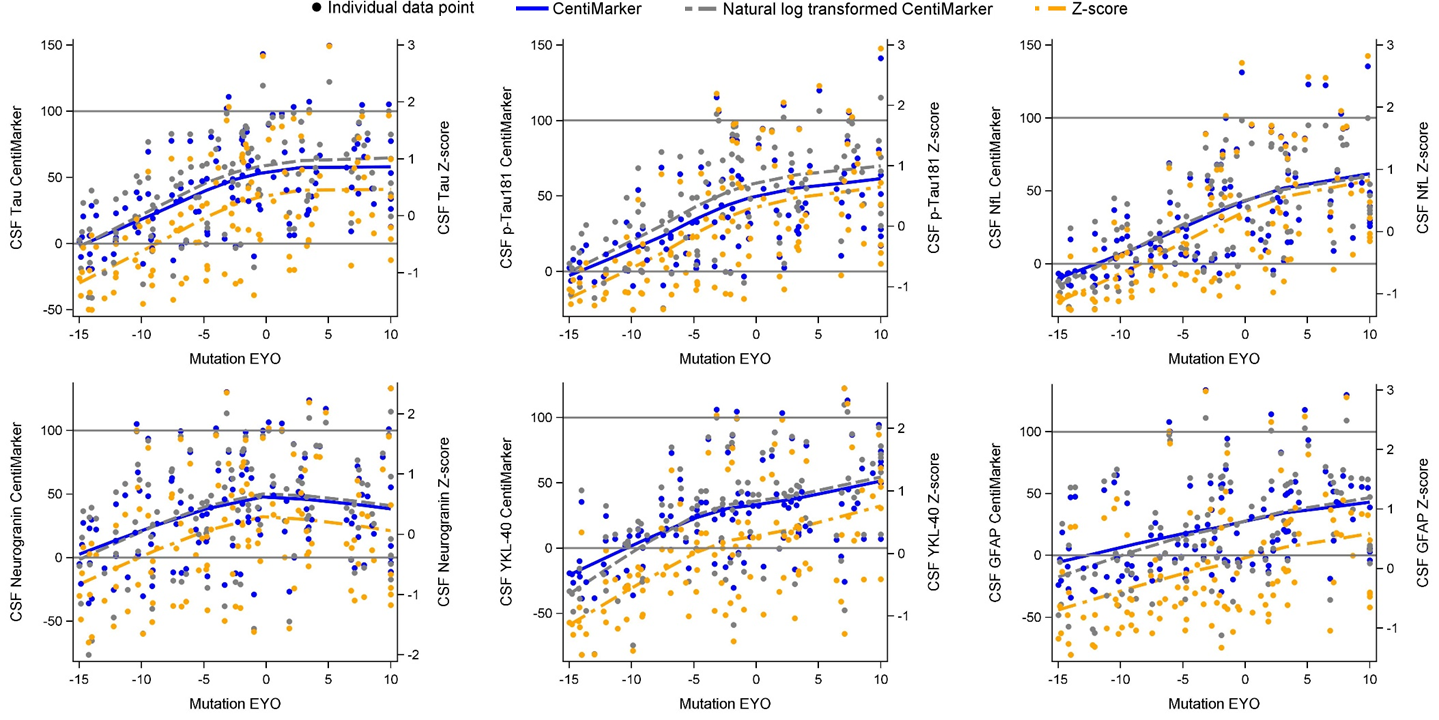
**

**Supplemental Figure 1:** Comparison of standardized values from three different methods: CentiMarkers, Natural Log-transformed CentiMarkers, and Z-scores. Natural Log-transformed CentiMarkers were calculated based on the natural log-transformed values, while z-scores were computed using the baseline mean and standard deviation of the mutation carriers. The mutation carriers included only the baseline data from the treatment groups and all the data from the placebo group in the DIAN-TU-001 trial. The x-axis represents the Estimated Years to Symptom Onset (EYO) based on mutation information, with zero indicating the expected onset of symptoms, covering a range of 25 years to represent disease progression. Dots represent individual data points.

**
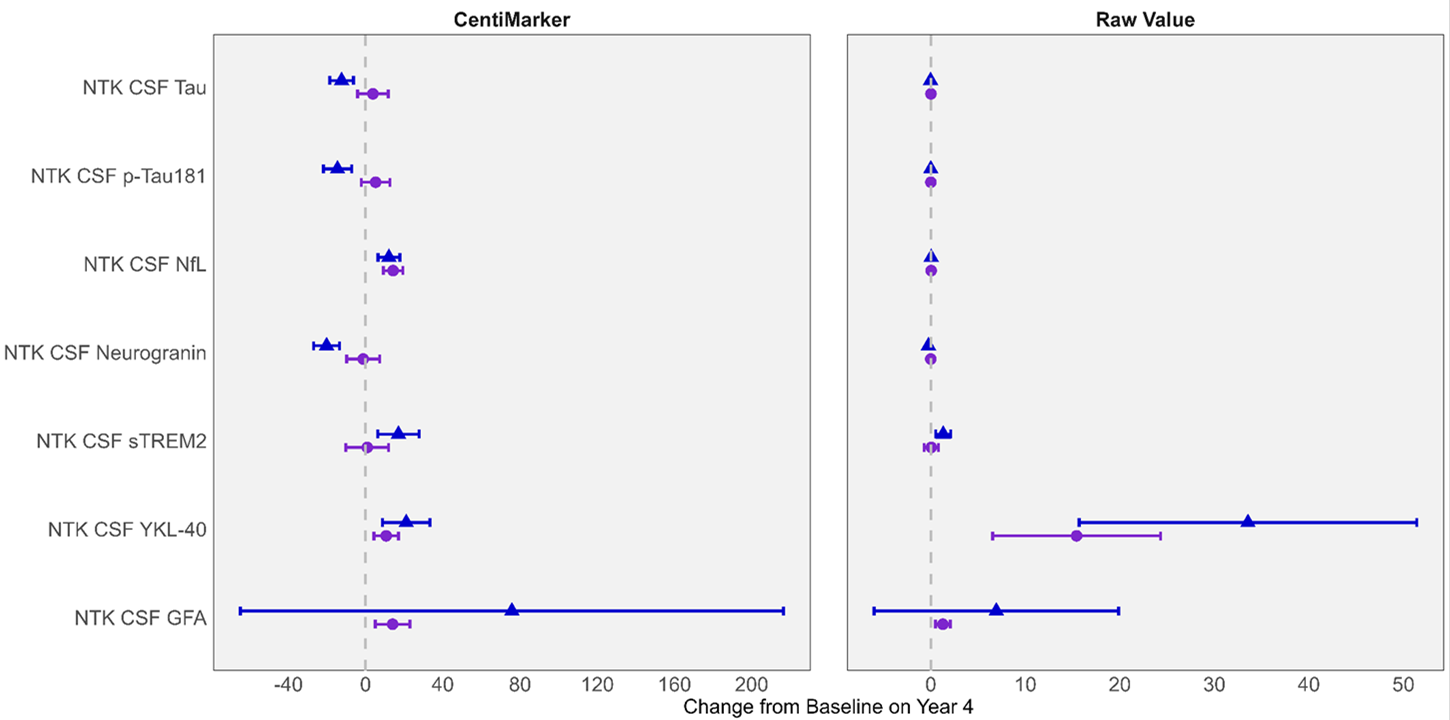
**

**Supplement Figure 2**: Utilizing CentiMarkers facilitates the interpretation and comparison across biomarkers by converting them to a similar scale, while using raw values is more difficult to compare as units are different and not scaled to disease ranges. Estimated mean change from baseline in CentiMarkers with 95% confidence intervals for the treatment and shared placebo groups using MMRM analyses in the DIAN-TU-001 trial of gantenerumab. These results demonstrate the magnitude of disease normalization compared to normal (CM 0) vs. fully abnormal (CM 100) states. Raw values are in the unit of ng/mL


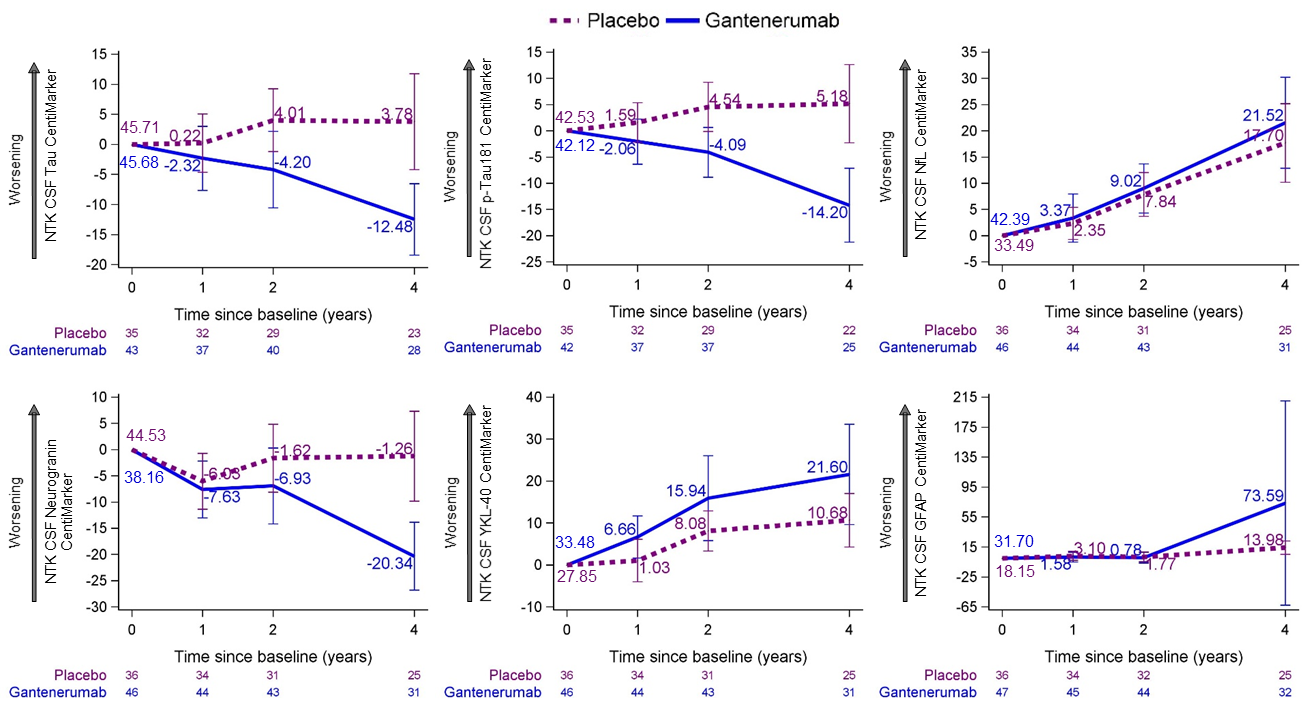


**Supplemental Figure 3**: Estimated mean change from baseline in CentiMarkers with 95% confidence intervals for the gantenerumab and the placebo groups using MMRM analyses in the DIAN-TU-001 trial. These results demonstrate the magnitude of disease normalization compared to normal (CM 0) vs. fully abnormal (CM 100) states.
